# Organic Acid-Induced Structural Modifications Improve Melt-Stretch Properties and Mouthfeel of Plant-Based Cheese Alternatives

**DOI:** 10.3390/foods14213724

**Published:** 2025-10-30

**Authors:** Can Xu, Lijun Liu, Jia Liu, Fayin Ye, Cuilan Fang, Lin Lei

**Affiliations:** 1 College of Food Science, Southwest University, Chongqing 400715, China; litchi0302@email.swu.edu.com (C.X.); lijun002@e.ntu.edu.sg (L.L.); fye@swu.edu.cn (F.Y.); 2 Guizhou Academy of Agricultural Sciences, Guiyang 550006, China; mcgrady456@163.com; 3 Chongqing Key Laboratory of Speciality Food Co-Built by Sichuan and Chongqing, Chongqing 400715, China; 4 Jiulongpo Center for Disease Control and Prevention, Chongqing 400039, China

**Keywords:** zein-based cheese, plasticizer, rheological and tribological properties, secondary structures, organic acid

## Abstract

Developing plant-based cheeses that replicate the melt-stretch property of dairy cheese remains challenging because plant proteins are brittle and thermally unstable. We hypothesized that combining acetic acid (plasticizer) with lactic or citric acid (acidulants) would enhance the melt-stretch behavior of zein-based cheese alternatives. Small-angle X-ray scattering analysis showed that the zein network formed semi-crystalline lamellar structures. Acetic acid promoted smaller, more uniformly distributed oil droplets and a denser protein matrix. Fourier transform infrared spectroscopy and rheological results indicated appropriate combinations of acetic acid and acidulants, which increased protein structural disorder and network flexibility. Melt-stretch and tribological evaluations further confirmed that the L/A2 formulation (lactic acid: acetic acid = 2:1) achieved a stretch length of 19.5 cm and a transition speed of 0.38 mm/s, closely resembling Cheddar cheese (26.2 cm; 0.38 mm/s), and outperforming a commercial plant-based Violife cheese (2.5 cm; 597.16 mm/s). This study provides practical guidance for designing appealing, allergen-conscious dairy alternatives.

## 1. Introduction

Replicating the melt-stretch properties of traditional dairy cheese in plant-based formats remains a major technological challenge because plant proteins are brittle and thermally unstable [1,2]. As plant-based diets expand for health, environmental, and ethical considerations, consumer demand for dairy-free cheese alternatives continues to rise. Commercial plant-based cheeses typically combine highly saturated vegetable oils, plant proteins (e.g., soy, pea, potato, wheat gluten, nuts), starches, stabilizers, emulsifiers, acidulants, and preservatives to mimic dairy cheese texture and flavor. However, the inclusion of common allergens such as nuts, soy, and gluten has accelerated interest in allergen-conscious formulations that avoid these ingredients [1].

Dairy cheese exhibits distinctive thermoreversible viscoelastic properties above ~40 °C, primarily due to milk fat melting and disruption of non-covalent interactions within the casein network [2]. Efforts to replicate these properties often use coconut or palm oils and starches, yet reproducing the casein structure–function relationships remains challenging. Zein, a maize-derived prolamin-rich storage protein, is a promising structuring protein for plant-based food matrices due to its amphiphilic nature and ability to form a viscoelastic network upon hydration and heating above its glass transition temperature (*T_g_*, ~45 °C) [3,4,5,6,7]. Zein self-assembles into hierarchical networks primarily via non-covalent interactions; nevertheless, zein-based structures are typically brittle and mechanically unstable, restricting their application in stretchable food systems. Additionally, the relatively high *T_g_* of hydrated zein restricts melt flow at moderate temperatures [4,6,8].

Plasticization is a common strategy to enhance zein flexibility by lowering *T_g_* and weakening intermolecular interactions [8,9,10,11,12]. Previous studies have demonstrated that acetic acid effectively disrupts zein intermolecular interactions and lowers *T_g_* more efficiently than ethanol, owing to its superior solvation and protonation capabilities [10]. However, the pungent odor of acetic acid limits consumer acceptance. Thus, food-grade organic acids such as lactic acid (21 CFR184.1061) [13] and citric acid (21 CFR184.1033) [14], both Generally Recognized as Safe (GRAS), are attractive alternatives given their established roles in dairy products for acidification, stabilization, and flavor. Recent studies have demonstrated that these acids could effectively reduce brittleness in zein-based matrices while maintaining favorable melt-stretch characteristics [8,15]. Nevertheless, systematic evaluations of food-grade organic acid plasticization effects in zein-based cheese alternatives remain limited.

Our previous work [7] demonstrated that incorporating highland barley *β*-glucan (HBG) into zein-based cheese improved rheological characteristics toward traditional Cheddar cheese. HBG (0–30%) refined the microstructure by producing smaller and more uniformly distributed oil droplets within the protein network, increasing stretchability (7.76–16.47 cm) beyond a commercial plant-based Violife cheese (6.72 cm) and approaching Cheddar (23.69 cm). Oscillatory temperature sweeps showed a crossover between G′ and G″ for Cheddar at approximately 60 °C, indicating a transition from viscoelastic solid to liquid. However, no such transition occurred in the zein-based cheeses with HBG, highlighting the need for zein plasticization to improve flowability [7].

Hence, this study aimed to systematically evaluate the effects of acetic, lactic, and citric acids on the melt-stretch properties of zein-based cheese alternatives, examining their rheological, tribological, and textural behavior. Comparative analyses against commercial dairy Cheddar and a popular plant-based cheese brand (Violife) were performed to assess the viability of zein-HBG systems as functional, allergen-conscious cheese substitutes suitable for broader consumer acceptance.

## 2. Materials and Methods

### 2.1. Materials

HBG (≥85% purity, molecular weight 2.0 × 10^5^ Da) and maize zein (≥95% purity, comprising 75–85% *α*-zein, 10% *β*-zein, and 5% *γ*-zein, molecular weight of 3.0 × 10^4^ Da) were sourced from Xian Sinuote Biotechnology Co., Ltd., Xian, China. Coconut oil, high-oleic sunflower oil (≥80% oleic acid), maize starch, and tapioca starch were bought from local suppliers. Food-grade citric acid, lactic acid, and acetic acid were purchased from the Jia He Xu Ri online marketplace and Shuangliu District Oushi Trade Department (Chengdu, China), respectively. For comparative analysis, Swissmooh medium Cheddar cheese and Violife medium Cheddar-style plant-based cheese (Cheddar flavor block) were obtained from an online supermarket. Nutritional labeling indicated that the Cheddar cheese contained approximately 31% fat, 0% carbohydrate, and 29% protein by weight, while the Violife plant-based cheese contained 24% fat, 20% carbohydrates, and 1.3% protein by weight. The primary ingredients in Violife plant-based cheese included coconut oil, modified starch, and ground sunflower kernels. All other chemicals and reagents used were of analytical grade.

### 2.2. Zein-Based Cheese Formulation

Zein-based cheese samples were prepared using our previous formulation with modifications [7]. Briefly, the formulation consisted of 30% zein, 5% HBG, 13.33% starch (a blend of maize starch and tapioca starch in a 1:2 ratio), 6.67% oil (composed of 25% high-oleic sunflower oil and 75% coconut oil to mimic the fatty acid profile of Cheddar cheese), and 30% distilled water (wt/wt). The organic acid composition was adjusted by decreasing lactic and citric acid concentrations while increasing acetic acid, maintaining a constant total organic acid content of 15% (wt/wt) (Table S1). Zein, starch, HBG, and powdered lactic or citric acid were initially mixed thoroughly before being combined with the lipid mixture, acetic acid, and distilled water at 60 °C in a thermostatic water bath. The resulting dough was homogenized by continuous stirring at 1500 r/min for 5 min using a multifunctional mixer (CX-306662, CHIGO, Hangzhou, China). Subsequently, the homogenized mixture was poured into molds, cooled to room temperature, and stored at 4 °C for 24 h before analysis. Zein-based cheese containing lactic acid, alone or in combination with acetic acid at ratios of 3:1, 2:1, and 1:1 (lactic acid: acetic acid), were designated as L, L/A3, L/A2, and L/A1, respectively. Similarly, samples formulated with citric acid alone or in combination with acetic acid at the same ratios were denoted as C, C/A3, C/A2, and C/A1, respectively.

### 2.3. Confocal Laser Scanning Microscope (CLSM) Imaging

The microstructures of samples were examined using a CLSM (LSM800, Carl Zeiss, Jena, Germany) at 200× magnification [7]. Briefly, samples were sectioned into 20 μm slices using a freezing microtome (Leica CM1850, Leica Microsystems, Nussloch, Germany). Sections were stained by applying 50 μL of a dye mixture containing Nile red (0.1% in 1,2-propanediol) and Fast Green FCF (0.1% in water) at a 1:3 ratio. The samples were then incubated at 4 °C for 15 min, followed by gentle washing three times with distilled water. Afterwards, the samples were stained with 40 μL of Fluorescent Brightener 28 (0.2% in distilled water, wt/wt) for 15 min at 4 °C and washed gently three times with distilled water. The stained samples were mounted onto glass slides and covered with glass coverslips for imaging. Nile red, Fast Green FCF, and Fluorescent Brightener 28 were excited using 488 nm, 633 nm, and 405 nm Ar lasers, respectively. Images were acquired and processed using Imaris Viewer software (version 9.5.1, Bitplane AG, Zurich, Switzerland).

### 2.4. Small Angle X-Ray Scattering (SAXS) Determination

SAXS measurements were conducted at Beamline BL16B1 of the Shanghai Synchrotron Radiation Facility (SSRF, Shanghai, China). The experimental setup involved a distance of 1900 mm between the sample and a Pilatus 1MF detector. Each sample was exposed to 10 s, with an electron energy of 10 keV and a beam current of 180 mA. The silver behenate standard was used to calibrate the sample to detector distance. Prior to tests, an incident X-ray photon of wavelength 0.124 nm was scattered through an empty sample holder to correct SAXS data. The 2D diffraction patterns obtained were analyzed using SGTools v1.0 software. Guinier analysis of the scattering data was performed using BioXTAS RAW v2.3.1 functions. The recorded q range spanned from 0.01 to 0.03 nm. Structural changes were monitored at 25 °C, and all SAXS data were presented in log*I*(q) versus log(q) plots. The scattering vector was calculated according to Equation (1) [16], where 2θ represented the scattering angle and λ was the X-ray wavelength.

*q* = (4π/*λ*) sin (2θ), (1)

The lamellar and fractal structures of each sample were quantified using the fractal dimension. The scattering patter from a fractal object followed a power law according to Equation (2) [16], where I denoted the scattering intensity and q represented the scattering vector.

*I∝ q^α^*, (2)

The fractal characteristics of the samples were characterized by the exponent *α*, which corresponded to the slope of the regression line in the double logarithmic SAXS curve. An exponent range of −4 < *α* < −3 was associated with the surface fractal, indicating that the scattering can be described as reflection from the surface or interface. In contrast, a range of −3 < *α* < −1 corresponded to mass fractal, where the density profile of the scattering object exhibited a self-similar nature. The surface fractal dimension (*D_s_*) and mass fractal dimension (*D_m_*) were determined using Equation (3) and Equation (4) [16], respectively.

*D_m_* = −*α*, (3)

*D_S_* = *6* + *α*, (4)

### 2.5. Fourier Transforms Infrared Spectroscopy (FTIR) Determination

All samples were analyzed using FTIR spectroscopy (Spectrum 100, Perkin-Elmer, Waltham, MA, USA). Measurements were recorded at room temperature over the range of 4000 cm^−1^ to 600 cm^−1^, with 16 scans and a resolution of 4 cm^−1^ at 25 °C. Spectra were collected against an air background, and background subtraction was applied to all spectra. Data analysis was performed using OMNIC software (version 8.2, Thermo Fisher Scientific, Waltham, MA, USA). Comparative analyses were also conducted on zein and HBG powders. The secondary structures of all samples containing zein were calculated by deconvoluting the secondary amide I region (1600–1700 cm^−1^) using Gaussian curve fitting in Peak Fit v4.12 software, leveraging the advantages of the secondary derivative for separating amide I components without an arbitrary factor input.

### 2.6. Moisture, Color, Water-Holding Capacity (WHC), and Free Oil Release

The moisture content of each sample was analyzed according to the Chinese National Standard GB 5009.3–2016 [17]. Color parameters, lightness (L*), redness (a*), and yellowness (b*) were quantified using an Ultrascan PRO Hunterlab colorimeter (Hunter Lab, Reston, VA, USA). WHC was assessed following our previous method [7]. Briefly, 2 g of each sample was placed in a 10 mL centrifuge tube and centrifuged at 10,000× *g* for 15 min at 4 °C. The expelled water was removed and WHC was calculated according to Equation (5).

*WHC* (%) = (*W*_2_/*W*_1_) ×100%, (5)

*W*_1_ represented the weight of sample (g) before centrifugation, and *W*_2_ represented the weight of the sample (g) with water removed after centrifugation.

Free oil release was calculated using our previous method with modifications [7]. Samples (30 mm in diameter × 5 mm in thickness) were cut from the interior of the cheese and equilibrated at room temperature for 30 min. The sample was then placed onto filter paper within a glass Petri dish, heated in an oven at 90 °C for 20 min, and cooled at room temperature for 60 min before being photographed. The area corresponding to released oil absorbed onto the filter paper was quantified using Image J v1.54j software (National Institutes Health, Bethesda, MD, USA).

### 2.7. Analysis of Stretchability

Stretchability was assessed using a previous method with modifications [18]. Briefly, a square-shaped sample (2 cm in width × 1 cm in thickness) was cut from the interior region of the cheese and equilibrated at room temperature for 30 min. The sample was then heated in a water bath (HCJ-6E, Changzhou, China) at 90 °C for 10 min and subsequently vertically stretched by gently lifting one end using forceps. The rupture distance was recorded as a measure of stretchability.

### 2.8. Rheological Determination

Rheological measurements were carried out using a rotational rheometer (DHR2, TA Instruments, New Castle, DE, USA) following a slightly modified method [6]. Briefly, 5.0 g sample was placed on the lower plate and compressed to a thickness of 2500 µm using a 40 mm diameter parallel plate. Strain sweep testing was conducted at 60 °C (above the melting temperature of Cheddar cheese) over a strain amplitude ranging from *γ* = 0.01% to *γ* = 1000% at an angular frequency of *ω* = 3 rad/s. Temperature sweep tests were performed from 4 °C to 95 °C at a shear strain of *γ* = 0.01% and angular frequency of *ω* = 3 rad/s.

### 2.9. Measurement of Friction Behavior

Lubrication properties of samples were determined using three-balls-on plate tribo-rheometry (Discovery Hybrid Rheometer DHR-2, TA Instrument, New Castle, DE, USA). A rough plastic surface simulating the roughness and wettability characteristics of the human tongue was created using Transpore Surgical Tape 1527-2 (3M Health Care, Saint Paul, MN, USA) [19]. Prior to testing, samples were equilibrated at room temperature for 30 min, and 3 g of sample was used to fully cover the substrate surface. A constant normal force of 2 N, reflecting moderate oral processing forces, was applied. Lubrication performance was assessed by measuring friction coefficients at sliding velocities ranging from 0.01 rad/s to 50 mm/s at 37 °C. Friction curves were generated by plotting the friction coefficients against sliding speeds, with maximum friction coefficients derived from these plots.

### 2.10. Texture Profile Analysis

Texture analysis was conducted by a Texture Analyzer (TA. XT Plus, Stable Micro System, Surrey, UK) using a cylindrical P/36R probe (30 mm diameter) according to our previous method at 4 °C, 37 °C, and 60 °C, respectively [7]. Each sample underwent two consecutive compressions to 75% of its original height (25% strain) at a constant speed of 1.5 mm/s for the pre-test, test, and post-test phases. A 5 s rest interval was applied between compressions, and an automatic trigger force of 5 g was used to initiate contact.

### 2.11. Statistical Analysis

For each formulation, six independent batches were prepared for textural analysis, while all other tests were performed in triplicate using three independent batches. Data were expressed as the mean ± standard deviation (SD). Statistical analysis was analyzed by one-way analysis of variance (ANOVA) followed by Turkey’s multiple-range test using SPSS software (SPSS Inc., Chicago, IL, USA; Version 26.0). The significance was set at *p* < 0.05.

## 3. Results and Discussions

### 3.1. Characterization of Zein-Based Cheese

#### 3.1.1. Effect of Organic Acids on the Morphology of Zein-Based Cheese

**Figure 1:**
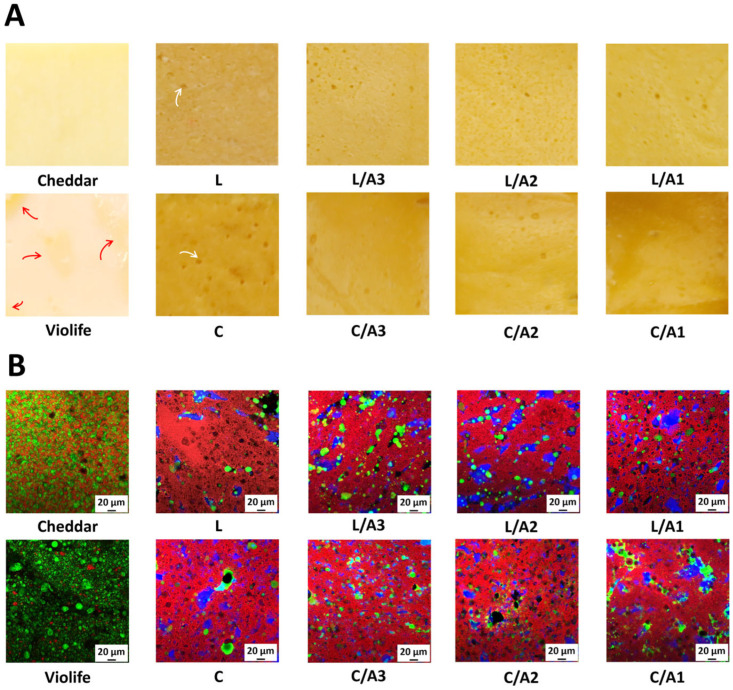
Visual appearance of surface (**A**) and microstructures (**B**) of Cheddar, Violife, and zein-based cheeses with different concentrations of organic acids. (**B**) Confocal laser scanning microscope images (scale bar: 20 μm) of all samples, where the fluorescent brightener (HBG), Fast Green FCF (zein), and Nile red (oil) are colored blue, red, and green, respectively. Red arrows indicate oil separation, while white arrows highlight pores. L, zein-based cheese with lactic acid; L/A3, zein-based cheese with lactic and acetic acids at a ratio of 3:1; L/A2, zein-based cheese with lactic and acetic acids at a ratio of 2:1; L/A1, zein-based cheese with lactic and acetic acids at a ratio of 1:1; C, zein-based cheese with citric acid; C/A3, zein-based cheese with citric and acetic acids at a ratio of 3:1; C/A2, zein-based cheese with citric and acetic acids at a ratio of 2:1; C/A1, zein-based cheese with citric and acetic acids at a ratio of 1:1.

Figure 1A illustrates the surface morphologies of Cheddar cheese, Violife cheese, and zein-based cheese. Cheddar and Violife cheese exhibited homogeneous surface structures. However, Violife cheese displayed incomplete starch-mediated oil emulsification with visible oil separation (red arrows) [7]. In contrast, L and C presented yellowish, rough, and porous surfaces (white arrows). Notably, adding acetic acid, particularly in the L/A2 and C/A3 formulations, reduced surface porosity, yielding a more continuous microstructure. Cross-sectional observations displayed similar trends (Figure S1).

Figure 1B presents the CLSM images of all samples, where the fluorescent brightener (HBG), Fast Green FCF (zein), and Nile red (oil) are colored blue, red, and green, respectively. Cheddar cheese exhibited a compact structure with irregularly shaped fat globules evenly dispersed within a continuous protein matrix. The non-spherical globule was attributed to mechanical shearing during processing, which disrupted the fat globule membrane and promoted partial coalescence during curd handling and pressing. In comparison, Violife cheese contained larger, spherical oil droplets embedded within the starch matrix (black areas) [20]. Zein-based cheese without acetic acid featured larger, polydisperse oil droplets (Figure 1B, L, and C). In contrast, L/A2 and C/A3 showed smaller, more uniformly distributed droplets, consistent with acetic acid plasticization increasing zein chain mobility. Mechanistically, acetic acid protonated and hydrated zein, promoting chain rearrangement and solubilization. The resulting increase in chain mobility exposed hydrophobic residues, strengthening zein–zein hydrophobic associations and yielding a more continuous protein matrix [21]. Consistent with prior CLSM observations of acetic-acid-plasticized zein, this cohesive network improved oil encapsulation and interfacial stabilization, producing smaller and more uniformly dispersed droplets [12,22].

#### 3.1.2. Effect of Organic Acids on Lamellar Features

**Figure 2:**
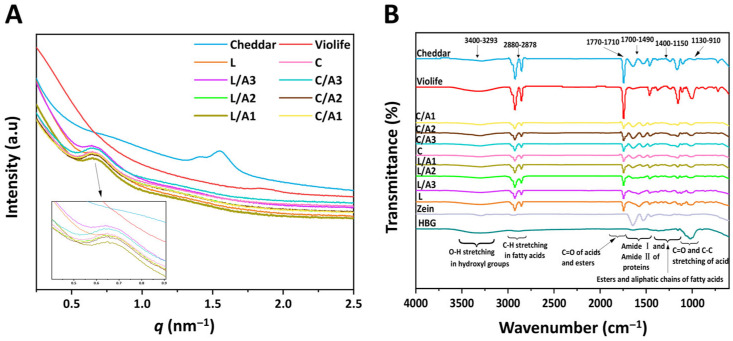
Small-angle X-ray scattering (**A**) and Fourier transform infrared spectroscopy (**B**) of Cheddar, Violife, and zein-based cheeses with different concentrations of organic acids. L, zein-based cheese with lactic acid; L/A3, zein-based cheese with lactic and acetic acids at a ratio of 3:1; L/A2, zein-based cheese with lactic and acetic acids at a ratio of 2:1; L/A1, zein-based cheese with lactic and acetic acids at a ratio of 1:1; C, zein-based cheese with citric acid; C/A3, zein-based cheese with citric and acetic acids at a ratio of 3:1; C/A2, zein-based cheese with citric and acetic acids at a ratio of 2:1; C/A1, zein-based cheese with citric and acetic acids at a ratio of 1:1.

Figure 2A presents the double logarithmic SAXS patterns of all samples. In Cheddar cheese, two prominent peaks at 1.40 nm^−1^ and 1.55 nm^−1^ were observed, indicative of crystalline phases of milk fat, corresponding to the lengths of dual aliphatic chains [23]. Violife cheese exhibited a scattering peak at 1.89 nm^−1^, signifying the lamellar liquid crystalline structure derived from coconut oil [24]. In comparison, zein-based cheese displayed scattering peaks between 0.58 nm^−1^ and 0.76 nm^−1^, associated with the semi-crystalline lamellar structures of maize/tapioca starch [25]. The SAXS data revealed α ranging from −3 to −1, indicating a mass fractal nature of the scattering sources within each sample. The fractal dimension (*D_m_*) quantifies structural compactness (Table 1). Values of *D_m_* = 1–2 reflect predominantly “surface-like” arrangements, whereas *D_m_* approaching 3 reflect densely packed 3D networks [16]. The observed *D_m_* values suggested predominantly surface-like structures in all samples. Notably, formulations combining lactic and acetic acids (*D_m_*, 1.24–1.58) were comparable to Cheddar (*D_m_*, 1.57) yet less compact than Violife (*D_m_*, 1.86), which showed the highest compactness among all samples. Acetic acid plasticized zein by partially disrupting interchain hydrogen bonding and hydrophobic contacts, increasing chain mobility, and enabling a more continuous, compliant fractal network. In combination with the acidulants, this converted the zein–starch matrix from a brittle assembly to a cohesive, deformable viscoelastic solid [12].

#### 3.1.3. Effect of Organic Acids on FTIR Spectra

The FTIR spectra of all samples are depicted in Figure 2B, displaying spectral features consistent with our previous findings [7]. Cheddar cheese exhibited typical FTIR spectra at 3700–900 cm^−1^, with bands arising from peptide bonds and the casein secondary structure as well as lipid and carbohydrate components. A broad band at 3700–3200 cm^−1^ indicated -OH stretching of the hydroxyl group. Aliphatic C-H stretching of fatty acids appeared at 3000–2800 cm^−1^, while carbonyl vibrations of lipids/esters were evident near 1750–1650 cm^−1^. Protein amide bands dominated 1650–1450 cm^−1^ (amide I/II), bands assigned to lipid methylene bending and C-O/C-O-C vibrations of esters and fatty acid chains were observed at the ester, and fat chains of fatty acids were represented at 1460–1150 cm^−1^. The 1200–900 cm^−1^ region was characteristic of polysaccharides (C=O and C-O stretching) [26]. Relative to Cheddar, Violife (plant-based) showed attenuated amide I/II features (1650 = 1450 cm^−1^) consistent with minimal protein, and enhanced polysaccharide-associated bands in the 1200–900 cm^−1^ region were due to higher starch content [27]. Zein showed typical protein spectral bands (1650–1450 cm^−1^), whereas HBG showed the expected polysaccharide spectral bands (1200–900 cm^−1^). Among the zein-based cheese analogues, spectra were broadly similar, with no new bands; only subtle intensity changes and minor shifts were observed across amide and carbohydrate regions. Notably, the O-H stretching envelope (3700–3200 cm^−1^) was broader in zein-based samples than in Cheddar or Violife, indicating increased hydrogen bonding within the complexes and contributions from intra-/intermolecular hydrogen bonding stretching [28].

The distribution of secondary structures in samples containing zein within the Amide I bands included a very low-frequency *β*-sheet (1615–1625 cm^−1^), low-frequency *β*-sheet (1620–1640 cm^−1^), random coil (1640–1648 cm^−1^), *α*-helix structure (1650–1660 cm^−1^), *β*-turn (1660–1675 cm^−1^), and high-frequency *β*-sheet (1675–1690 cm^−1^) (Table 2). Zhang et al. found that acetic acid transformed zein from a particulate aggregate into a viscoelastic network and shifted its secondary structure toward an increased low-frequency *β*-sheet with a reduced *α*-helix [12]. In our study, relative to native zein, the lactic acid sample (L) showed a higher proportion of the low-frequency *β*-sheet (22.4% → 39.1%) and lower *α*-helix (26.5% → 20.1%), whereas the citric acid sample (C) also increased the low-frequency *β*-sheet (26.9%) with the *α*-helix no longer detectable. In contrast, when acetic acid was combined with lactic acid, L/A3 and L/A2 exhibited lower proportions of the low-frequency *β*-sheet compared to native zein (Table 2, *p* < 0.05). The discrepancies were likely due to the distinct polarities of organic acids and their influence on zein protonation. Consequently, the formation of salt bridges involving protonated basic residues (Arg, Lys, His) of zein can vary with acid identity, leading to different plasticization outcomes [10,29].

Overall, compared to native zein, formulations L/A3, L/A2, C, and C/A3 exhibited a marked decrease in ordered secondary structures (*β*-sheet and *α*-helix) and an increase in disordered conformations (random coil and *β*-turn) (Table 2, *p* < 0.05). Greater disordered structures contributed to increased network flexibility [12]. The contents of disorder conformation followed C (64.0) > C/A3 (62.1) > L/A3 (51.4) > L/A2 (48.4) > zein (44.4), indicating that the plasticization magnitude depended on the acid type and mixing ratio [8,9,12,15].

### 3.2. Functional Properties of Zein-Based Cheese

#### 3.2.1. Effect of Organic Acids on Moisture, Color, WHC, and Free Oil Release

Due to the incorporation of yellowish sunflower oil, zein-based cheese exhibited lower L* values but higher a* and b* values compared to both Cheddar and Violife cheese (Table 1), consistent with the visual observations in Figure 1A. Both Cheddar and zein-based cheese demonstrated higher WHCs than Violife (Table 1), likely due to the protein networks formed by casein and zein, which restrict water loss [7,10]. Among all samples, Cheddar released the greatest amount of free oil, consistent with its highest fat content (Table 1). Within the zein formulations, citric acid produced lower free-oil release than lactic acid, plausibly because its tri-carboxylic functionality enabled stronger hydrogen bonding with zein, promoted hydrophobic exposure, and enhanced lipid encapsulation via zein–starch interactions [22].

#### 3.2.2. Effect of Organic Acids on Stretchability

Stretchability, a key property of cheese products, reflects the ability of protein network to maintain integrity under thermal and mechanical stress. Melted Cheddar cheese exhibited significantly greater stretchability (26.2 cm) than Violife (2.5 cm) (Table 1). In our previous study, a formulation containing 25% zein with 10% HBG exhibited functional cheese-like properties [7], while Mattice and Marangoni reported similar characteristics using 30% zein with 0.7% xanthan gum [6]. However, the stretchability in both systems remained limited, reaching 16.47 cm and 12.5 cm, respectively. The present results showed that the acid composition critically influenced zein network flexibility and melt-stretch behavior (Table 1). The L/A2 formulation achieved 19.5 cm stretchability, approaching Cheddar cheese. In contrast, L, L/A3, and L/A1 showed lower stretchability (9–16.5 cm), indicating suboptimal ratios and incomplete plasticization. C (35.2 cm) and C/A3 (22.8 cm) displayed excessive softness, while C/A2 (9.4 cm) and C/A1 (7.5 cm) were sticky and less stretchable. These observations were consistent with the protein secondary structural changes (Table 2), where C and C/A3 exhibited higher proportions of disorder secondary structures (>60%), likely contributing to softness and extensibility. Conversely, C/A2 and C/A1 showed more ordered structures (~35%), restricting chain mobility and stretch. Acetic acid was effective as a plasticizer, while lactic/citric acids introduced reversible hydrogen-bonded/ionic associations that stabilized a deformable network. At excessive acidity, over-plasticization weakened network integrity and reduced extensibility [8].

#### 3.2.3. Effect of Organic Acids on Oscillatory Strain Sweeps

**Figure 3:**
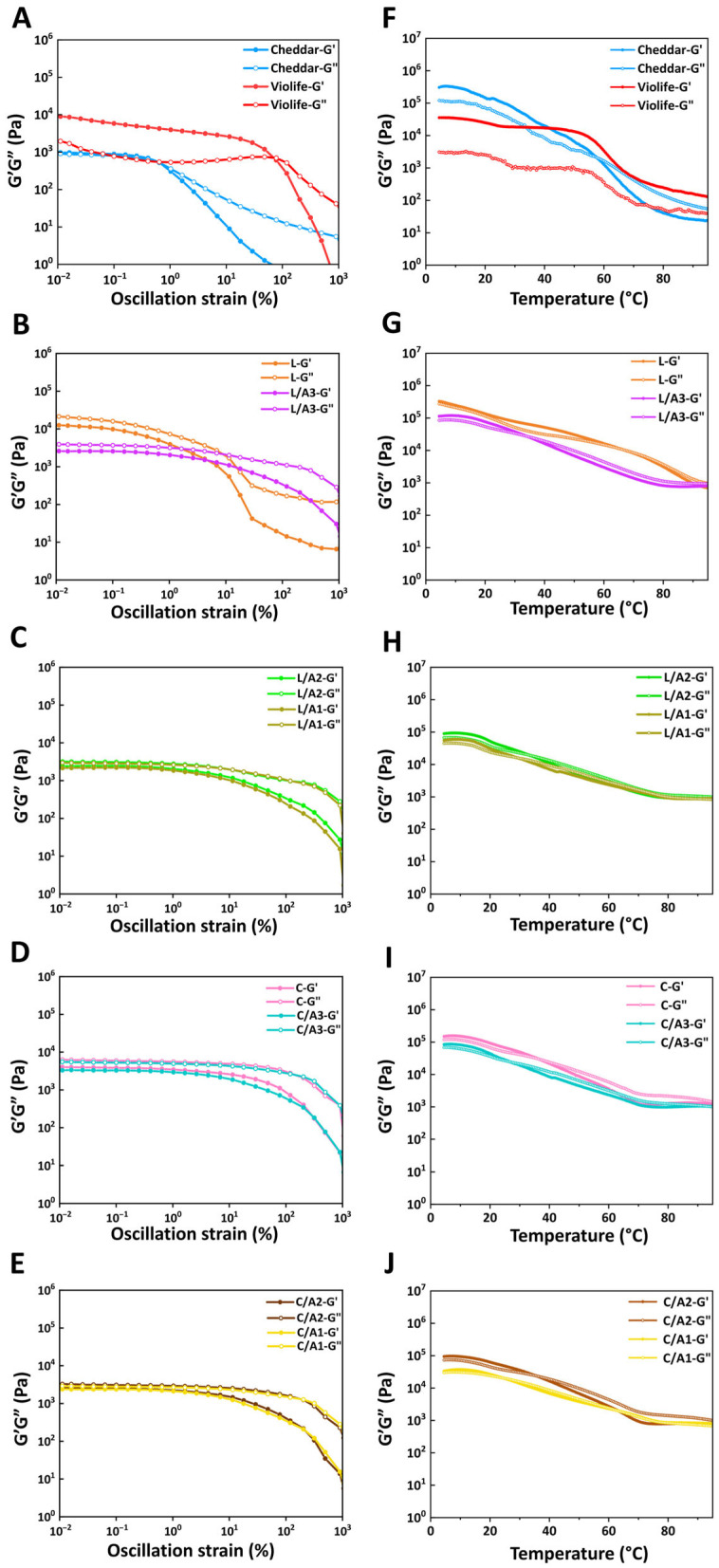
Oscillatory amplitude sweeps (**A**–**E**) and oscillatory temperature sweeps (**F**–**J**) of Cheddar, Violife, and zein-based cheeses with different concentrations of organic acids. L, zein-based cheese with lactic acid; L/A3, zein-based cheese with lactic and acetic acids at a ratio of 3:1; L/A2, zein-based cheese with lactic and acetic acids at a ratio of 2:1; L/A1, zein-based cheese with lactic and acetic acids at a ratio of 1:1; C, zein-based cheese with citric acid; C/A3, zein-based cheese with citric and acetic acids at a ratio of 3:1; C/A2, zein-based cheese with citric and acetic acids at a ratio of 2:1; C/A1, zein-based cheese with citric and acetic acids at a ratio of 1:1.

Figure 3A–E show the storage (G′) and loss modulus (G″) versus shear strain, capturing the linear viscoelastic region (LVR), defined as the strain range, over which G′ varies by <5% [30]. Both Cheddar cheese and Violife exhibited solid-like gel behavior within LVR (G′ > G″). Compared to Cheddar, Violife, rich in starch, demonstrated significantly higher G″ values at low strain levels and a markedly greater gap between G′ and G″ values (Figure 3A). In addition, Cheddar cheese reached the crossover point of G′ and G″ (~1.2%) earlier than Violife cheese (~77.5%), indicating that the starch-based network of Violife remained elastic over a wider strain range [6,7]. For zein systems, L (Figure 3B) exhibited higher G′ and G″ values than C (Figure 3D) at *γ* = 0.01%, consistent with L being less stretchable than C (Table 1). Upon adding organic acids, zein-based formulations exhibited G″ consistently exceeding G′ within the LVR (Figure 3B–E), indicating viscous-dominated responses attributable to organic acid plasticization [12,21].

#### 3.2.4. Effect of Organic Acids on Oscillatory Temperature Sweeps

Figure 3F–J illustrate the thermo-mechanical effects of organic acids on zein networks during temperature scanning. All samples, except for Violife, exhibited a viscoelastic solid-to-liquid transition upon heating (G′ > G″→G′ < G″), indicating progressive weakening of the protein network [6]. Cheddar crossed at ~60 °C (Figure 3F). For zein systems, adding acetic acid consistently lowered both G′ and G″ values compared to those formulated solely with lactic acid (L) or citric acid (C) (Figure 3G–J). The crossover temperatures followed L (~64 °C) > C (~38 °C) > L/A3 (~35 °C) > C/A2 and L/A2 (~32.3 °C) > L/A1 and C/A3 (~30.2 °C) > C/A1 (~25.2 °C). These crossovers occurred near the effective glass transition regime for zein, indicating that acetic acid facilitated glassy-to-rubbery transitions during heating [12,21]. In contrast, Violife primarily consisted of starch and fat, maintained highly elasticity (G′ > G″) and exhibited only slight softening from 4 °C to 60 °C (Figure 3F) Although zein-based cheese did not fully replicate the melting behavior of Cheddar cheese, their thermal profiles were notably closer to Cheddar than to Violife.

#### 3.2.5. Effect of Organic Acids on Tribological Measurement

**Figure 4:**
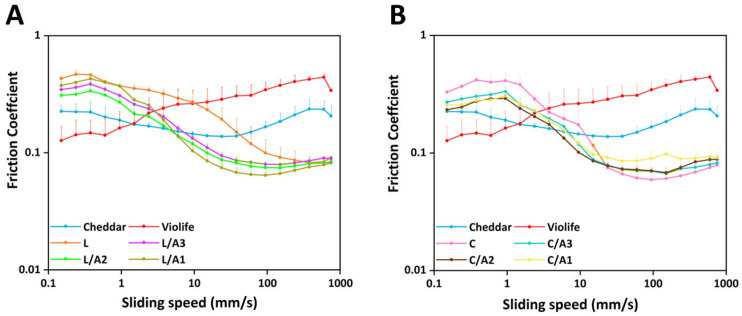
Friction coefficient (**A**,**B**) of Cheddar, Violife, and zein-based cheeses with different concentrations of organic acids. L, zein-based cheese with lactic acid; L/A3, zein-based cheese with lactic and acetic acids at a ratio of 3:1; L/A2, zein-based cheese with lactic and acetic acids at a ratio of 2:1; L/A1, zein-based cheese with lactic and acetic acids at a ratio of 1:1; C, zein-based cheese with citric acid; C/A3, zein-based cheese with citric and acetic acids at a ratio of 3:1; C/A2, zein-based cheese with citric and acetic acids at a ratio of 2:1; C/A1, zein-based cheese with citric and acetic acids at a ratio of 1:1.

Figure 4A,B depict the tribological behavior of all samples. Cheddar cheese exhibited a classical Stribeck curve (Figure S2), with a notable reduction in friction values above 597.16 mm/s (Table 3), indicative of casein network breakdown. Conversely, Violife exhibited a continuously rising friction trend across all tested speeds until structural failure occurred above 597.16 mm/s (Table 3), likely due to its high starch and ground sunflower kernels [31]. Zein-based cheese displayed tribological curves generally resembling those of Cheddar. However, at low to medium sliding speeds (0.10–3.77 mm/s), Cheddar demonstrated the lowest friction values, attributable to its high fat content, greater free-oil release, and uniformly dispersed fat globules (Figure 1B), which promoted interfacial film formation and enhanced lubrication [19]. At high sliding speeds (>10 mm/s), zein-based cheeses outperformed Cheddar in lubrication, exhibiting lower friction coefficients. This improvement was ascribed to the presence of acetic acid, which disrupted inter-chain hydrogen bonding, enhanced zein chain mobility, and enhanced lubricating film formation [12].

To further compare the impact of acid combinations on tribological behavior, the maximum friction coefficient value (*f*_1_) and transition velocity (*v*_1_)—indicative of lubrication film onset—are detailed in Table 3. The *f*_1_ values inversely correlate with perceived oral smoothness. Higher friction reflects poorer lubrication, often perceived as reduced creaminess or increased resistance during mastication. Among all formulations, L/A2 exhibited the most favorable tribological profile, achieving the lowest *f*_1_ value (0.34) and a *v*_1_ (0.38 mm/s) close to that of Cheddar (0.22, 0.38 mm/s). All lactic acid-based formulations (L, L/A3, L/A2, and L/A1) exhibited similarly low *v*_1_ values (~0.38 mm/s), significantly outperforming the citric acid-based counterparts (*v*_1_, 0.95 mm/s). This suggested that lactic acid synergized more effectively with acetic acid to facilitate earlier lubrication and smoother oral processing.

#### 3.2.6. Effect of Organic Acids on Texture Qualities

**Figure 5:**
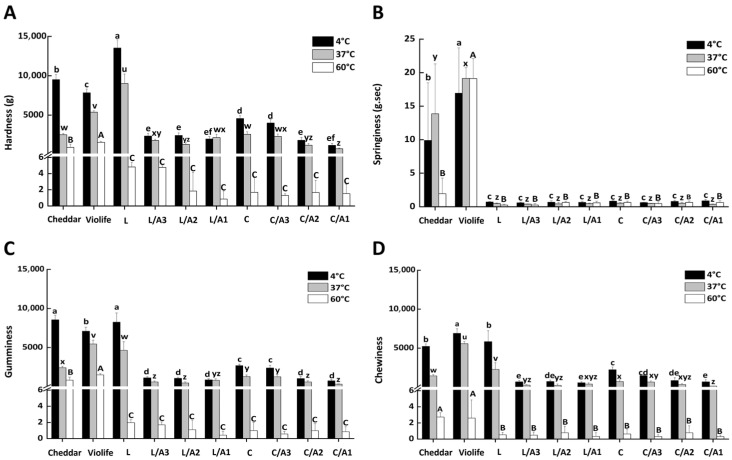
Texture profile parameters at 4 °C, 37 °C, and 60 °C of Cheddar, Violife, and zein-based cheeses with different concentrations of organic acids. (**A**) Hardness; (**B**) Springiness; (**C**) Gumminess; (**D**) Chewiness. L, zein-based cheese with lactic acid; L/A3, zein-based cheese with lactic and acetic acids at a ratio of 3:1; L/A2, zein-based cheese with lactic and acetic acids at a ratio of 2:1; L/A1, zein-based cheese with lactic and acetic acids at a ratio of 1:1; C, zein-based cheese with citric acid; C/A3, zein-based cheese with citric and acetic acids at a ratio of 3:1; C/A2, zein-based cheese with citric and acetic acids at a ratio of 2:1; C/A1, zein-based cheese with citric and acetic acids at a ratio of 1:1. Bars with different letters indicate statistically different (*p* < 0.05), where 4 °C samples (a–f), 37 °C (u–z) and 60 °C (A–C) analyzed separately.

The effects of organic acids on hardness, springiness, chewiness, and gumminess of zein-based cheese are presented in Figure 5. Textural analyses were conducted at 4 °C, 37 °C, and 60 °C to capture the temperature-dependent transitions in structure. As expected, increases in temperature progressively decreased hardness, gumminess, and chewiness, reflecting the melting and thermal softening of casein/zein networks (Figure 5A,C,D) [6,7]. At 37 °C, hardness ranked Violife > Cheddar > zein-based cheese, consistent with the tribological behavior observed at higher sliding speeds (Figure 4). The plasticizing effect of acetic acid was particularly evident in the lactic acid-based formulations. Relative to the lactic acid-only (L) sample, adding acetic acid reduced hardness, chewiness, and gumminess by 76–86%, 83–90%, and 83–91%, respectively. In citric acid-based systems, the corresponding reductions versus the citric-only sample (C) were 10–72%, 1–76%, and 8–83% (Figure 5A,C,D). The discrepancy likely reflected the distinct polarity and protonation capacity of lactic versus citric acid, which modulated zein protonation and network formation, thereby altering chain mobility.

## 4. Conclusions

This study showed the types and ratios of organic acids that governed the microstructure and melt-stretch behavior of zein-based cheese analogues. Using acetic acid as a plasticizer with lactic or citric acid as acidulants, the protein conformation/packing, oil droplet dispersion, and viscoelastic/tribological responses were studied. The mixed-acid L/A2 formulation (lactic: acetic = 2:1) achieved stretchability and lubrication-regime transitions approaching Cheddar and exceeding a commercial plant-based control (Violife). FTIR and SAXS revealed increased protein disorder and a more flexible network in mixed-acid systems, consistent with greater apparent chain mobility. In summary, these results identified L/A2 as a practical route to couple reduced-fat design with dairy-like melt-stretch. This work focused on physicochemical and functional properties, while sensory attributes (flavor, aroma, color) and targeted nutritional fortification were not assessed. Future studies should implement sensory testing and explore additions (e.g., sea salt, Cheddar flavor, calcium phosphate, *β*-carotene, and vitamin B_12_) to further narrow remaining gaps with conventional dairy cheese.

## Figures and Tables

**Table 1:** Effect of organic acids on SAXS data and functional characteristic indicators of zein-based cheese.

	Cheddar	Violife	L	L/A3	L/A2	L/A1	C	C/A3	C/A2	C/A1
α	−1.57 ± −0.08	−1.86 ± 0.00	−1.58 ± 0.04	−1.48 ± −0.13	−1.24 ± −0.06	−1.38 ± −0.26	−1.16 ± 0.02	−1.16 ± 0.00	−1.22 ± 0.01	−1.24 ± 0.04
*D_m_* (nm)	1.57 ± 0.08	1.86 ± 0.00	1.58 ± 0.04	1.48 ± 0.13	1.24 ± 0.06	1.38 ± 0.26	1.16 ± 0.02	1.16 ± 0.00	1.22 ± 0.01	1.24 ± 0.04
L*	72.8 ± 0.5 ^b^	81.2 ± 0.1 ^a^	64.0 ± 3.2 ^c^	57.3 ± 1.2 ^de^	58.3 ± 0.6 ^d^	58.9 ± 0.8 ^d^	55.1 ± 1.2 ^e^	44.5 ± 1.2 ^g^	50.5 ± 0.8 ^f^	43.8 ± 1.9 ^g^
a*	2.2 ± 0.2 ^e^	4.9 ± 0.1 ^d^	8.7 ± 1.3 ^abc^	7.3 ± 2.2 ^c^	7.6 ± 0.4b ^c^	8.0 ± 0.5 ^abc^	9.4 ± 0.9 ^a^	9.2 ± 0.3 ^ab^	9.7 ± 0.5 ^a^	10.0 ± 0.4 ^a^
b*	24.4 ± 0.3 ^e^	25.9 ± 0.1 ^de^	38.0 ± 3.0 ^ab^	33.4 ± 8.3 ^bc^	40.2 ± 0.6 ^a^	31.6 ± 3.3 ^cd^	40.9 ± 1.4 ^a^	33.4 ± 2.6 ^bc^	37.4 ± 0.9 ^abc^	31.4 ± 2.1 ^cd^
WHC (%)	97.8 ± 3.7 ^a^	93.3 ± 2.8 ^b^	99.7 ± 0.1 ^a^	97.0 ± 4.6 ^ab^	99.7 ± 0.1 ^a^	99.7 ± 0.0 ^a^	99.4 ± 0.2 ^a^	99.6 ± 0.3 ^a^	99.5 ± 0.2 ^a^	99.6 ± 0.4 ^a^
FOR (cm^2^)	32.1 ± 1.0 ^a^	5.2 ± 0.8 ^d^	11.9 ± 32.3 ^b^	7.3 ± 0.5 ^c^	7.6 ± 0.6 ^c^	7.2 ± 0.2 ^c^	5.3 ± 0.2 ^d^	4.2 ± 0.4 ^d^	4.0 ± 0.6 ^d^	3.6 ± 0.2 ^d^
Stretch (cm)	26.2 ± 2.9 ^b^	2.5 ± 0.4 ^e^	9.0 ± 2.3 ^d^	16.5 ± 1.6 ^c^	19.5 ± 3.0 ^c^	10.2 ± 1.5 ^d^	35.2 ± 5.8 ^a^	22.8 ± 1.8 ^b^	9.4 ± 1.6 ^d^	7.5 ± 1.8 ^d^

L, zein-based cheese with lactic acid; L/A3, zein-based cheese with lactic and acetic acids at a ratio of 3:1; L/A2, zein-based cheese with lactic and acetic acids at a ratio of 2:1; L/A1, zein-based cheese with lactic and acetic acids at a ratio of 1:1; C, zein-based cheese with citric acid; C/A3, zein-based cheese with citric and acetic acids at a ratio of 3:1; C/A2, zein-based cheese with citric and acetic acids at a ratio of 2:1; C/A1, zein-based cheese with citric and acetic acids at a ratio of 1:1; *α*, the power-law exponent derived from fitting small-angle X-ray scattering data; *D_m_*, the mass fractal dimension; WHC, water-holding capacity; FOR, free oil release. Stretch, stretchability. Different letters in the same row indicate significant differences (*p* < 0.05).

**Table 2:** Effect of organic acids on protein secondary structures of zein-based cheese.

Secondary Structure (%)	Zein	L	L/A3	L/A2	L/A1	C	C/A3	C/A2	C/A1
Very low-frequency *β*-sheet	10.9 ± 0.0 ^b^	35.3 ± 3.8 ^a^	12.9 ± 0.1 ^b^	6.6 ± 0.7 ^c^	10.1 ± 1.1 ^b^	11.4 ± 0.1 ^b^	13.0 ± 2.7 ^b^	11.1 ± 0.1 ^b^	10.6 ± 0.0 ^b^
Low-frequency *β*-sheet	22.4 ± 0.2 ^de^	39.1 ± 1.7 ^a^	14.3 ± 0.6 ^f^	20.6 ± 0.1 ^e^	24.2 ± 0.8 ^cd^	26.9 ± 0.9 ^b^	27.8 ± 2.3 ^b^	26.7 ± 0.0 ^b^	26.1 ± 0.3 ^bc^
Random coil	29.3 ± 0.1 ^d^	33.5 ± 1.2 ^c^	38.4 ± 0.8 ^a^	35.4 ± 0.7 ^bc^	33.7 ± 0.7 ^c^	36.5 ± 0.1 ^ab^	35.5 ± 2.9 ^bc^	35.8 ± 0.0 ^bc^	35.1 ± 0.4 ^bc^
*α*-helix structure	26.5 ± 0.3 ^d^	20.1 ± 1.2 ^e^	34.3 ± 1.9 ^a^	31.0 ± 0.3 ^b^	29.7 ± 0.8 ^bc^	—	—	29.0 ± 0.1 ^c^	28.9 ± 0.3 ^c^
*β*-Turn	15.0 ± 0.1 ^b^	7.3 ± 1.7 ^d^	13.1 ± 0.5 ^c^	13.0 ± 0.3 ^c^	12.4 ± 0.8 ^c^	27.4 ± 1.2 ^a^	26.6 ± 1.0 ^a^	—	—
High-frequency *β*-sheet	6.7 ± 0.1 ^c^	—	—	—	—	9.1 ± 0.2 ^b^	10.1 ± 0.6 ^a^	8.9 ± 0.0 ^b^	9.9 ± 0.4 ^a^
Ordered conformation	55.6 ± 0.1 ^c^	59.2 ± 0.5 ^b^	48.5 ± 1.4 ^e^	51.6 ± 0.4 ^d^	53.9 ± 0.3 ^c^	36.0 ± 1.0 ^f^	37.9 ± 2.2 ^f^	64.6 ± 0.0 ^a^	64.8 ± 0.4 ^a^
Disordered conformation	44.4 ± 0.1 ^d^	40.8 ± 0.5 ^e^	51.4 ± 1.4 ^b^	48.4 ± 0.4 ^c^	46.1 ± 0.3 ^d^	64.0 ± 1.0 ^a^	62.1 ± 2.2 ^a^	35.4 ± 0.0 ^f^	35.1 ± 0.4 ^f^

L, zein-based cheese with lactic acid; L/A3, zein-based cheese with lactic and acetic acids at a ratio of 3:1; L/A2, zein-based cheese with lactic and acetic acids at a ratio of 2:1; L/A1, zein-based cheese with lactic and acetic acids at a ratio of 1:1; C, zein-based cheese with citric acid; C/A3, zein-based cheese with citric and acetic acids at a ratio of 3:1; C/A2, zein-based cheese with citric and acetic acids at a ratio of 2:1; C/A1, zein-based cheese with citric and acetic acids at a ratio of 1:1. Different letters in the same row indicate significant differences (*p* < 0.05).

**Table 3:** Effect of maximum coefficient of friction value (*f*_1_) at transition speed (*v*_1_) for Cheddar cheese, Violife cheese, and zein-based cheeses.

Various Samples	*v*_1_ (mm/s)	*f*_1_
Cheddar	0.38	0.22 ± 0.05
Violife	597.16	0.44 ± 0.03
L	0.38	0.46 ± 0.00
L/A3	0.38	0.38 ± 0.02
L/A2	0.38	0.34 ± 0.05
L/A1	0.38	0.43 ± 0.02
C	0.95	0.41 ± 0.07
C/A3	0.95	0.33 ± 0.03
C/A2	0.95	0.29 ± 0.02
C/A1	0.95	0.31 ± 0.04

L, zein-based cheese with lactic acid; L/A3, zein-based cheese with lactic and acetic acids at a ratio of 3:1; L/A2, zein-based cheese with lactic and acetic acids at a ratio of 2:1; L/A1, zein-based cheese with lactic and acetic acids at a ratio of 1:1; C, zein-based cheese with citric acid; C/A3, zein-based cheese with citric and acetic acids at a ratio of 3:1; C/A2, zein-based cheese with citric and acetic acids at a ratio of 2:1; C/A1, zein-based cheese with citric and acetic acids at a ratio of 1:1.

## Data Availability

The original contributions presented in the study are included in the article/Supplementary Materials. Further inquiries can be directed to the corresponding authors.

## Supplementary Materials

The following supporting information can be downloaded at https://www.mdpi.com/article/10.3390/foods14213724/s1, Table S1: Zein-based cheese compositions, Figure S1: Visual appearance of cross-section; Figure S2: A Stribeck curve and its three regimes.

## Abbreviations

The following abbreviations are used in this manuscript:

HBG	Highland barley β-glucan
*T_g_*	Glass transition temperature
*D_m_*	Mass fractal dimension
*D_s_*	Surface fractal dimension

## References

[1] Grossmann L., McClements D.J. (2021). The science of plant-based foods: Approaches to create nutritious and sustainable plant-based cheese analogs. Trends Food Sci. Technol..

[2] Lamichhane P., Kelly A.L., Sheehan J.J. (2018). Structure-function relationships in cheese. J. Dairy Sci..

[3] Zhang Y., Xu M., Zhang X., Hu Y., Luan G. (2022). Application of zein in gluten-free foods: A comprehensive review. Food Res. Int..

[4] Mattice K.D., Marangoni A.G. (2020). Evaluating the use of zein in structuring plant-based products. Curr. Res. Food Sci..

[5] Schober T.J., Bean S.R., Boyle D.L., Park S.-H. (2008). Improved viscoelastic zein-starch doughs for leavened gluten-free breads: Their rheology and microstructure. J. Cereal Sci..

[6] Mattice K.D., Marangoni A.G. (2020). Physical properties of plant-based cheese products produced with zein. Food Hydrocoll..

[7] Liu L., Huang G., Li S., Meng Q., Ye F., Chen J., Ming J., Zhao G., Lei L. (2023). Replacement of fat with highland barley β-glucan in zein-based cheese: Structural, rheological, and textual properties. Food Chem. X.

[8] Sly A.C., Taylor J., Taylor J.R.N. (2014). Improvement of zein dough characteristics using dilute organic acids. J. Cereal Sci..

[9] Mattice K.D., Marangoni A.G. (2020). Functionalizing zein through antisolvent precipitation from ethanol or acetic acid. Food Chem..

[10] Li Y., Li J., Xia Q., Zhang B., Wang Q., Huang Q. (2012). Understanding the Dissolution of α-Zein in Aqueous Ethanol and Acetic Acid Solutions. J. Phys. Chem. B.

[11] King B.L., Taylor J., Taylor J.R.N. (2016). Formation of a viscoelastic dough from isolated total zein (α-, β- and γ-zein) using a glacial acetic acid treatment. J. Cereal Sci..

[12] Zhang X., Gao M., Zhang Y., Dong C., Xu M., Hu Y., Luan G. (2022). Effect of plasticizer and zein subunit on rheology and texture of zein network. Food Hydrocoll..

[13] U.S. Government Publishing Office 21 CFR 184.1061—Lactic Acid. https://www.ecfr.gov/current/title-21/chapter-I/subchapter-B/part-184/subpart-B/section-184.1061/.

[14] U.S. Government Publishing Office 21 CFR 184.1033—Citric Acid. https://www.ecfr.gov/current/title-21/chapter-I/subchapter-B/part-184/subpart-B/section-184.1033/.

[15] Wei L., Dou M., Zhang W., Xu X., Chen H., Zhang Z. (2024). Characterization of zein-based films plasticized with deep eutectic solvents and their use in the preservation of harvested mango fruit. Food Hydrocoll..

[16] Suzuki T., Chiba A., Yarno T. (1997). Interpretation of small angle X-ray scattering from starch on the basis of fractals. Carbohydr. Polym..

[17] (2016). Determination of Moisture Content in Food.

[18] Chailangka A., Leksawasdi N., Seesuriyachan P., Ruksiriwanich W., Sommano S.R., Jantanasakulwong K., Rachtanapun P., Castagnini J.M., Barba F.J., Phimolsiripol Y. (2023). Improving vitamin D stability and antioxidant activity in imitation mozzarella cheese by conjugated cricket protein with fructooligosaccharide. LWT Food Sci. Technol..

[19] Khanal B.K.S., Bhandari B., Prakash S., Bansal N. (2020). Simulated oral processing, in vitro digestibility and sensory perception of low fat Cheddar cheese containing sodium alginate. J. Food Eng..

[20] Grasso N., Roos Y.H., Crowley S.V., Arendt E.K., O’Mahony J.A. (2021). Composition and physicochemical properties of commercial plant-based block-style products as alternatives to cheese. Future Foods.

[21] Zhang X., Xu M., Zhang Y., Li J., Wang J., Hu Y., Luan G. (2022). Effect of zein subunit and plasticizer on rheology and adhesion properties of zein-based adhesives. Ind. Crops Prod..

[22] Zhang Y., Wu F., Wang J., Xu M., Cao S., Hu Y., Luan G. (2024). Impacts of ethanol-plasticization and extrusion on development of zein network and structure of zein-starch dough. Food Chem..

[23] Zhang Y., Ma T., Zhang F., Guo W., Yu K., Yang C., Qu F. (2022). Multiple phase transitions and microstructural rearrangements shape milk fat crystal networks. J. Colloid Interface Sci..

[24] Mahamod W.R.W., Bakar N.A., Hashim N., Isnolamran N.H., Shamsudin S.A.J.M.J. (2021). The effect of virgin coconut oil content on the rheological profile of virgin coconut oil-based lamellar liquid crystal of mixed Tween 80: BRIJ 30 System. Malays. J. Microsc..

[25] Zhang Y., Junejo S.A., Zhang B., Fu X., Huang Q. (2022). Multi-scale structures and physicochemical properties of waxy starches from different botanical origins. Int. J. Biol. Macromol..

[26] Tarapoulouzi M., Pashalidis I., Theocharis C.R. (2024). Discrimination of Cheese Products Regarding Milk Species’ Origin Using FTIR, 1H-NMR, and Chemometrics. Appl. Sci..

[27] Warren F.J., Gidley M.J., Flanagan B.M. (2016). Infrared Spectroscopy as a Tool to Characterise Starch Ordered Structure—A Joint FTIR–ATR, NMR, XRD and DSC Study. Carbohydr. Polym..

[28] Jin B., Zhou X., Zheng Z., Liang Y., Chen S., Zhang S., Li Q. (2020). Investigating on the Interaction Behavior of Soy Protein Hydrolysates/*β*-Glucan/Ferulic Acid Ternary Complexes under High-Technology in the Food Processing: High Pressure Homogenization versus Microwave Treatment. Int. J. Biol. Macromol..

[29] Huo W., Wei D., Zhu W., Li Z., Jiang Y. (2018). High-elongation zein films for flexible packaging by synergistic plasticization: Preparation, structure and properties. J. Cereal Sci..

[30] Kasapis S., Bannikova A., Ahmed J., Ptaszek P., Basu S. (2017). Chapter 2—Rheology and Food Microstructure. Advances in Food Rheology and Its Applications.

[31] Lin S., Zhao J., Wang Z., Sun F., Shi J., Zhang J., Li L., Li M. (2024). Lubricating properties of starch-soybean lecithin compound gels using tribology analysis. LWT Food Sci. Technol..

