# Supplementary material for: Organic Acid-Induced Structural Modifications Improve Melt-Stretch Properties and Mouthfeel of Plant-Based Cheese Alternatives
Source: Foods. 2025 Oct 30;14(21):3724. doi: 10.3390/foods14213724 (PMC12607658; doi:10.3390/foods14213724)
Supplement: Supplementary file 1 [file foods-14-03724-s001.zip › foods-3920594-supplementary.pdf]

Supplementary data

Organic Acid-Induced Structural Modifications Improve Melt-Stretch Properties and Mouthfeel of Plant-Based Cheese Alternatives

Can Xu [1], Lijun Liu [1], Jia Liu [3], Fayin Ye[1, 4], Cuilan Fang [2,*] and Lin Lei [1, 4,*]

1 College of Food Science, Southwest University, Chongqing 400715, China

2 Jiulongpo Center for Disease Control and Prevention, Chongqing 400039, China

3 Guizhou Academy of Agricultural Sciences, Guiyang 550006, China

4 Chongqing Key Laboratory of Speciality Food Co-Built by Sichuan and Chongqing, Chongqing 400715, China

* Correspondence: fangcuilan@163.com (C.F); leilinsky@swu.edu.cn (L.L)

Table

Table S1. Zein-based cheese compositions

Component (%)	L	L/A3	L/A2	L/A1	C	C/A3	C/A2	C/A1
**Zein**	30	30	30	30	30	30	30	30
**HBG**	5	5	5	5	5	5	5	5
**Starch component**	13.33	13.33	13.33	13.33	13.33	13.33	13.33	13.33
**Oil component**	6.67	6.67	6.67	6.67	6.67	6.67	6.67	6.67
**Water**	30	30	30	30	30	30	30	30
**Lactic acid**	15	11.25	10	7.5	—	—	—	—
**Citric acid**	—	—	—	—	15	11.25	10	7.5
**Acetic acid**	—	3.75	5	7.5	—	3.75	5	7.5

L, zein-based cheese with lactic acid; L/A3, zein-based cheese with lactic and acetic acids at a ratio of 3:1; L/A2, zein-based cheese with lactic and acetic acids at a ratio of 2:1; L/A1, zein-based cheese with lactic and acetic acids at a ratio of 1:1; C, zein-based cheese with citric acid; C/A3, zein-based cheese with citric and acetic acids at a ratio of 3:1; C/A2, zein-based cheese with citric and acetic acids at a ratio of 2:1; C/A1, zein-based cheese with citric and acetic acids at a ratio of 1:1. HBG, highland barley *β*-glucan.

Figures

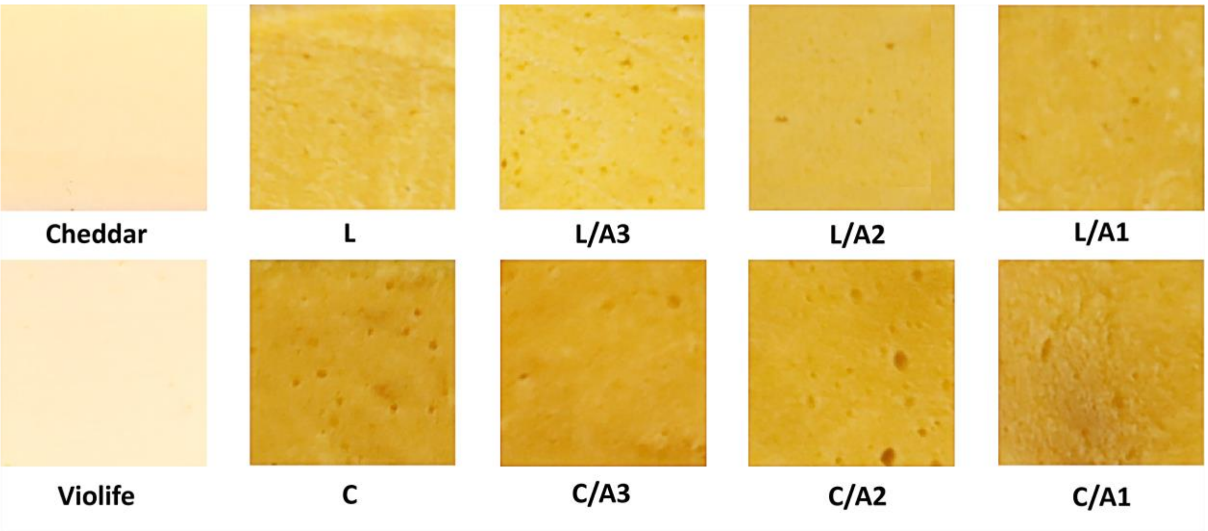

Figure S1. Visual appearance of cross-section of Cheddar, Violife, and zein-based cheeses with different concentrations of acidity regulators and processing aids. L, zein-based cheese with lactic acid; L/A3, zein-based cheese with lactic and acetic acids at a ratio of 3:1; L/A2, zein-based cheese with lactic and acetic acids at a ratio of 2:1; L/A1, zein-based cheese with lactic and acetic acids at a ratio of 1:1; C, zein-based cheese with citric acid; C/A3, zein-based cheese with citric and acetic acids at a ratio of 3:1; C/A2, zein-based cheese with citric and acetic acids at a ratio of 2:1; C/A1, zein-based cheese with citric and acetic acids at a ratio of 1:1. HBG, Highland barley β-glucan.

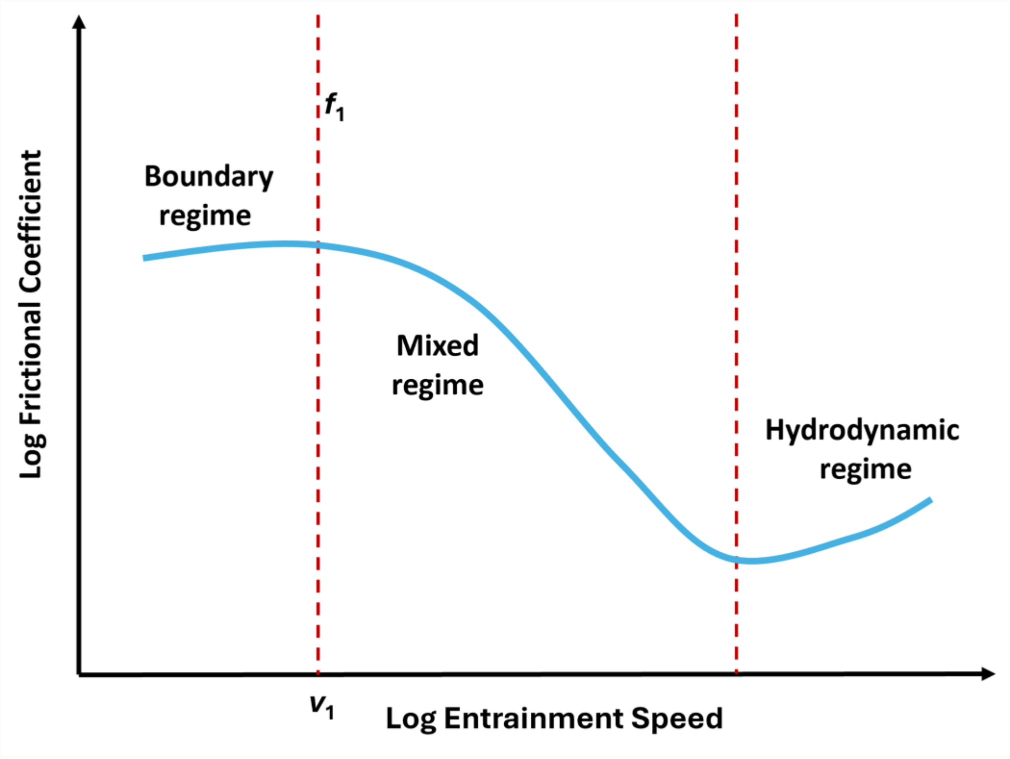

Figure S2. A Stribeck curve and its three regimes.
